# Patient family advisors’ perspectives on engagement in health‐care quality improvement initiatives: Power and partnership

**DOI:** 10.1111/hex.12633

**Published:** 2017-09-28

**Authors:** Donna Goodridge, Tanner Isinger, Thomas Rotter

**Affiliations:** ^1^ College of Medicine University of Saskatchewan Canada; ^2^ Queen's University Kingston ON Canada

**Keywords:** patient engagement, power, quality improvement, service user involvement

## Abstract

**Background:**

Engagement of the public in defining and shaping the organization and delivery of health care is increasingly viewed as integral to improving quality and promoting transparent decision making. Meaningful engagement of the public in health‐care reform is predicated on shifting entrenched power imbalances between health‐care systems and those it claims to serve.

**Objectives:**

To describe the expressions, forms and spaces of power from the perspectives of persons who participated as Patient/Family Advisors (PFAs) in Rapid Process Improvement Workshops (RPIWs) within Saskatchewan, Canada.

**Methods:**

Using a qualitative, interpretive approach, in‐depth interviews were conducted with a purposive sample of 18 PFAs who had participated in at least one RPIW over the past year. Deductive thematic analysis was informed by Gaventa's model of power.

**Results:**

Motivations for serving as a PFA included a sense of obligation to contribute to the improvement of a public system, recognition of their rights as citizens within a publicly funded system and an opportunity to openly express their concerns where previous encounters had been very negative. The invited spaces of the RPIWs were created by policymakers to accord visible power to PFAs. Participation resulted in PFAs gaining new insights into the structure and operations of the system, affirmation of their right to advocate and recognition of the potential to claim spaces of power as consumers. Advisement on specific health‐care initiatives using the vehicle of PFAs shaped and promoted new forms and spaces of power, representing one step in a very long road to full engagement of consumers in health care.

## 1 INTRODUCTION

The person‐centred health‐care reform agenda is characterized by increasing efforts to engage both patients and the public in defining and shaping the organization and delivery of health care.^1^, ^2^, ^3^ Patient engagement has been defined as the involvement of patients and their families, integrated alongside health professionals, to improve health and health‐care services.^4^ As a strategy to improve quality and promote decision‐making transparency,^5^, ^6^ the benefits of patient engagement are suggested to result from: capitalizing on the expertise of patients and their networks; allowing for more service choice for patients; enhancing responsiveness to changing user needs; and reducing cost and waste by identifying and addressing redundancies and overlap.^7^

While public involvement in health‐care reform can be promoted using a range of initiatives,^8^ implementation of strategies that promote meaningful patient engagement remains a challenge,^7^, ^9^ in part because of deeply entrenched power imbalances between patients, providers and policymakers.^10^ Informed by Gaventa's theory of power, the objectives of this qualitative study were to describe the expressions, forms and spaces of power from the perspectives of persons who participated as Patient/Family Advisors (PFAs) in quality improvement initiatives called Rapid Process Improvement Workshops (RPIWs) within the health‐care system of Saskatchewan, Canada. RPIWs are intensive quality improvement events that focus on a single problem in service delivery, identify the root cause and create solutions. PFAs serve as integral members of the team working to address these objectives.

## 2 BACKGROUND

Although health care putatively exists to meet the needs of patients, ideologies reflecting the vested interests of providers and funders have often resulted in hegemonic and paternalistic systems described as “coercive and controlling, without choices as to refusing care, what kinds of care may be considered, or who provides the care.”^11^ In conventional health‐care systems, power is vested in providers and funders while patients and families are accorded a passive role as beneficiaries of health‐care services. “User involvement is ‘in the gift’ of services, in that it is discretionary whether service‐users are invited to participate or under what conditions.”^11^

Burgeoning public mistrust^12^, ^13^, ^14^ and new demands for transparency and accountability^15^, ^16^ in health care have been catalysts for the development of new forms of partnership with those whom the system is intended to serve. This paradigm shift reflects growing support for the principles of democracy and consumerism within health care, which is marked by greater public involvement and enhanced accountability.^1^ Increasing recognition that health‐care services, unlike goods, are coproduced^7^ has led to widespread efforts to build effective partnerships and engage with patients, caregivers and the public with a view to incorporating their perspectives in health‐care redesign.

Efforts to engage the public in health‐care decision making are not without detractors. The goals, the nature and extent of activities intended to foster patient engagement are often decided by powerful gate‐keepers within the health‐care system. Although patients and families are increasingly called upon to act as coproducers of health care, sharing navigation is challenging when accountability for the overall system ultimately rests with professionals and bureaucrats. “It is neither possible nor desirable to share power and responsibility equitably between patients and professionals in all situations…[in some instances], the burden of responsibility…must fall disproportionately on health care professionals”.^7^ Tokenism, suboptimal quality of involvement and lack of resources for meaningful engagement of patients have also been noted as barriers to genuine engagement.^17^ Patient engagement initiatives have further been criticized as window‐dressing efforts by decision makers to legitimize foregone decisions when power imbalances are not actually re‐aligned.^11^ Critical examination of patient engagement initiatives, particularly in the area of mental health, has found relatively minimal impact in terms of addressing pre‐existing structural inequalities and making any differences to the patient experience.^18^, ^19^, ^20^, ^21^

## 3 THEORETICAL PERSPECTIVE

Gaventa developed a theoretical approach to examining power through his investigation of social dynamics in an impoverished Appalachian valley mining region.^22^ His theory was initially developed to help explain the reasons underlying the acquiescence and passive agreement of groups facing social oppression who were excluded from decision making in matters of key concern to them.^23^ Gaventa's theory holds promise for examining the role of patient engagement in health care, an arena in which patients and families have typically been excluded from participation and decision making, and, until recently, have generally complied and acquiesced to this status quo.

Gaventa described a three‐dimensional model of power^24^ (“the power cube”) comprised of levels, forms and spaces as a means to include the voices to marginalized groups in development initiatives. Power is conceptualized as having four expressions: “power over,” “power with,” “power to,” and “power within.” The forms of power include invisible, hidden and visible power. Depending on context and an individual's personal resources (eg, social advantage), conventional health‐care systems may be encountered primarily from the vantages of either invisible or hidden power. Invisible power refers to the “internalization of powerlessness,” with obfuscation of one's rights and interests as a result of the adoption of dominant ideologies, values and forms by the relatively powerless group themselves. “People may be unaware of their rights, their ability to speak out and may come to see various forms of power or domination over them as ‘natural’, or at least unchangeable.”^24^ The “culture of silence” resulting from internalization of oppression described by Freire^25^ aligns particularly well with the lived experience of disadvantaged patients in conventional health‐care system.

In contrast, activated citizens are aware of and able to articulate their grievances with conventional systems; they deal with predominantly hidden forms of power.^24^ The hidden power in health care exercised by governments, funders and providers creates barriers to public participation, excludes key issues from the public arena and controls politics from “backstage”.^24^ Creating a culture of person‐centred care involves an evolution towards Gaventa's^22^ third type of power. Visible forms of power provide access to the decision‐making arenas of organizations as neutral playing fields in which “those with grievances are able to articulate them in the formal decision‐making processes and participate fully in deliberations.”^24^

Within the power cube model, spaces create opportunities to enable different types of power.^24^ Decision‐making spaces not accessible outside a small circle of privileged people of power are considered closed, while invited spaces occur when citizens are invited to participate in decision making by authorities.^24^ Claimed/created spaces evolve organically when people share common issues or concerns and may be fluid depending on the issue. Those creating the space are likely to have power within it. Drawing upon this model, we submit an initial hypothesis that the inclusion of PFAs in quality improvement initiatives promotes visible power of the public by creating invited spaces.

Traditional health‐care decision‐making spaces have not been accessible outside of a small circle of privileged people of power (health‐care providers, bureaucrats) and are considered closed decision‐making spaces. Within the RPIW, decision makers deliberately constructed a novel and invited space that promotes the participation of users, patients and beneficiaries in decision making.^24^

## 4 METHODS

Informed by Gaventa's theory of power,^24^ an interpretive approach was used to describe the motivations, experience and self‐identified outcomes for persons who participated as Patient/Family Advisors (PFAs) in Rapid Process Improvement Workshops (RPIWs). Qualitative research designs informed by social theory can serve to orient researchers to concepts and process that they might not necessarily identify through inductive processes alone.^26^

### 4.1 Ethical approval

Ethical approval for this project (13‐294) was granted by the University of Saskatchewan Behavioural Research Ethics Board.

### 4.2 Setting and participants

Within the province of Saskatchewan, the large‐scale implementation of Lean in 2012 was introduced as a strategy by the provincial government to promote patient‐centredness and improve health‐care quality.^26^ Lean principles focus on ensuring that all processes add value to the “customer”; any processes or activities that do not add value are considered “waste.”^27^ RPIWs are intensive Lean activities lasting five consecutive days directed at eliminating waste within the health‐care system.^28^ Teams of PFAs, staff and leaders focus on a single problem in service delivery, identify the root cause and create solutions to be evaluated after 30, 60 and 90 days.^27^ Participants in each RPIW vary according to the nature of the event, but include front‐line providers, physicians, decision makers and staff from related departments. PFAs have been mandated as essential participants in RPIWs and must commit to attend the entire event. RPIWs cannot proceed without the participation of one or more PFAs.

PFAs are “individuals who have recent experiences with the Saskatchewan health‐care system as a patient or a family member. They volunteer their time to provide perspectives of patients and families to planning, and development, implementation and evaluation of policies and programs.”^29^ Honoraria and expenses are paid to PFAs according to prescribed rates. PFAs may be engaged in formal initiatives, such as working groups and RPIWs, or may serve informally as consultants on specific projects on an ad hoc basis.^28^ While PFAs were not initially involved in the design of the RPIW events, their on‐going feedback was incorporated into later iterations of the RPIWs.

A purposive sample of participants for this study was recruited through Kaizen Promotion Officers in health regions and by an invitation posted on the PFA Facebook page hosted by the Patient and Family Centred Care Network. The Kaizen Promotion Office provides leadership and support for strategic improvement initiatives. Eligibility criteria for this study consisted of participation as a PFA in at least one RPIW in the previous year.

Interviews were conducted in May and June 2016 with eighteen adult PFAs, with 17 being face to face and one by telephone due to geographic distance. Eleven participants were females. Six participants were aged 65 and older; eight were between 45 and 64 years of age; and four were less than 45 years old.

There was appropriate geographical representation within the sample. Ten participants lived in one of the two larger urban health regions in the province, with the remainder from smaller urban health regions. Eight PFAs were retired, two were employed full‐time, one was employed part‐time, and the remainder were not currently employed outside the home. Seven PFAs reported having been employed in health care in a variety of roles at some point in their working careers.

The sample comprised participants with a wide diversity of personal experiences as a patient or family member with the provincial health‐care system. Four of the participants reported having had very negative or catastrophic previous experiences, including permanent personal injury or the death of family members that were attributable to failings of the health‐care system. Five PFAs indicated their experiences with health care had been primarily positive, while the remainder reported both positive and negative experiences. Most (15) of the participants had participated in multiple RPIWs prior to the interviews.

### 4.3 Data collection

A semi‐structured interview guide was constructed to reflect broad themes from the patient engagement literature and the objectives of the project in face‐to‐face interviews. Seven open‐ended questions dealt with: (i) previous experiences with health care, (ii) experience with RPIWs, (iii) motivations to participate as a PFA in the RPIW and the recruitment process, (iv) characteristics of effective PFAs, (v) type and extent of training provided, (vi) perspectives on participation in the RPIW(s), and (vii) preferences for future citizen engagement in health‐care quality improvement. Interviews with 18 PFAs lasted between 30 and 95 minutes and were audio‐taped with participant's informed consent.

### 4.4 Data analysis

Interviews were transcribed verbatim and analysed using a deductive thematic analysis approach to identify, analyse and report patterns (themes) within the data.^30^ Two of the researchers independently reviewed, annotated and coded each of the transcripts and then collaboratively created initial codes for the data.^31^ The collated codes were reviewed and sorted according to overarching themes.^30^ The transcripts were carefully re‐examined for relevant data relating to each theme and additional data entered into the coding framework. Two of the researchers jointly reviewed, defined and named to accurately and thoroughly represent the data and ensure consensus.^30^ The final form of each theme was constructed guided by Gaventa's^24^ power cube theory (expressions, spaces and forms of power), with the addition of a new theme drawn from the data (outcomes for the PFAs). Charts summarizing the data from all 18 participants were created for each of the themes. The final themes for coding were as follows: (i) expressions of power within the health‐care system (eg, encounters with providers; consumer orientation; opportunities for patients and families to have their voices heard), (ii) forms of power (eg, invisible power, visible power), (iii) the RPIW as a new space of power (eg, perceived changes in status, quality of interactions), and (v) outcomes of participation as a PFA (eg, empowerment, reactions to participation).

## 5 RESULTS

### 5.1 Expressions of power within the health‐care system

Most PFAs reported one or more encounters within the health‐care system in the past that had been clearly disrespectful and dehumanizing, emphasizing the common occurrence of having been in situations in which providers exercised unwelcome “power over” patients and family members. PFAs’ descriptions of their previous encounters as recipients of care within the health care, irrespective of whether the experiences were positive or negative, reflected their positions as actors without power or status in the rigid hierarchy of the system. The *“doctor's in charge, or doctor thinks he's god, and nurse thinks she's the right hand, and what they say goes and you have nothing to say as a patient.”* The power wielded by health‐care providers allowed for the perceived lack of accountability and transparency with respect to medical error and suspicions of “cover‐ups” within the health‐care system for some PFAs. *“[Providers] don't want to be called on [the mistake] …There were huge mistakes made.”*

PFAs clearly believed that patient and families were generally considered “guests” in the health‐care system, and as guests, exercised little power. *“We don't belong to the groups…we are not part of the system.”* The common perception that the health‐care system was “owned” by providers, and not patients or the public, was exemplified in the following quote: *“Everybody else [professionals on the RPIW team] has a vested interest; I mean they've got some piece in this whole thing and us patient advisors are very much outsiders.”* Some PFAs identified that patients and families should be entitled to the power and respect accorded to a consumer, rather than a passive recipient of care, within the health‐care system. “*You're not treated like a customer at all. You're treated as an annoyance.”*

Participating as a PFA offered new opportunities to share “power with” the existing health‐care system in designing strategies to improve services and outcomes. “Power with” strategies are based upon finding common ground amongst diverse interests and building collective strength to build bridges to promote equitable relations.^22^ Seven of the PFAs volunteered to participate after reading advertisements, while the remainder were approached by health‐care staff. All PFAs recognized that maintaining a status quo which excluded patients and the public would not produce the transformations required. *“Our healthcare system needs changing, and it's not going to change if we don't say what we want, and we don't speak about our experiences.”*

Motivations to participate as a PFA were often predicated upon acting on expressions of “power to” and “power within.” “Power to” can be described as the unique opportunities of every individual to shape his or her life and world, which reflects agency and opens up the possibilities of joint action, whereas “power within” refers to the capacity to imagine and have hope for a better world.^22^

A number of PFAs remarked on the value of personal learning opportunities inherent in the RPIW and recognized these were novel opportunities to have power given to them, to gain “insider knowledge” not typically available to patients and families. *“Information is power, and so I always like the opportunity to be able to do that kind of thing.”*

PFAs acknowledged their power as citizens within a publicly funded health‐care system. Expressions of “power to” were often based in the language of rights. *“I have a right to come into a hospital to get healthy, not to get sicker.”* Demand to exercise this power, however, was tempered by an acknowledgement that other factors required consideration in making changes. *“I'm not **his** patient, he's **my** doctor. So I should I have a right to say my decision on my healthcare, to a certain degree.”*

Regardless of whether they had had positive or negative experiences with the health‐care system in the past, most participants were ultimately motivated to participate from the stance of “power within”—a sense of obligation to contribute to the betterment of a public service as the driving force to volunteer for this initiative. *“I'm being a responsible citizen.” “I realize that I can't fix them, but you're always kind of obliged to find a way to point out this and that, and try and help.”* Empathy for others and altruism was another facet of obligation: *“I just feel for all the people that don't have family or an advocate. How do they push to be heard?”* Gratitude was also a consistent motivator for those who had good experiences. *“The system's been good to me and my family. It's a rewarding feeling to give something back.”* The primary concern of most PFAs about participating in the RPIW related to the relatively large commitment of time. “*The time away from my family and away from my job. I took vacation to go*.”

### 5.2 Forms of power

Invisible power, in which people are unaware of their rights and their ability to speak out, was not a prominent feature in the discourse of PFAs in this study, although there was some evidence that the chance to participate in the RPIWs was novel. Several PFAs were initially cynical about becoming engaged in the RPIW and wondered *“Was I was going to be the token patient that nobody ever listens to?”* Feelings of apprehension about participation were voiced by some PFAs, especially prior to their first RPIW, and where there was less certainty about what to expect and on what basis the PFAs would be accorded power within the group. “*Would I be able to match these guys intellectually in the discussions and make sense?…whether I would be able to understand them.”*

The invitation to participate as a PFA created a visible form of power in which access was provided to a health‐care decision‐making arena. This visible power was enacted primarily as the responsibility to give voice to the concerns and issues of patients and families by *“being articulate… taking the lesson from their own experience and applying it broadly.”* “*If I don't speak up, then the opportunity to bring that patient family voice is lost.”* One PFA suggested that they had a regulatory function: “*Be the watchman just in case the technical persons get carried away, and they drift away from the objective. You know, the patient.”*

PFAs felt responsible to remind providers of the importance of a holistic approach and a respectful attitude, both within the RPIW event and in the clinical arena. “*There is a very social and clinical aspect of healthcare, but often a clinician will forget.”* “*What I think is really, really important to remind providers that when the patient and family come, that they're to listen and speak with them with respect.”*

### 5.3 The RPIW as a new space of power

Inclusion of PFAs in the RPIW created a novel form of invited space that disrupted pre‐existing power imbalances in the health‐care system and allowed for altered expressions and forms of power. The opportunity to share their stories with a captive audience of health‐care providers served as an important outcome of the RPIW for some PFAs. *“I was one of your patients and you goofed. You failed me real bad.”*

Traditional power imbalances between patients and providers were effectively flattened for the duration of the RPIW. Having the ability to contribute their voices in a forum in which high‐level health‐care executives participated was clearly astonishing and initially somewhat uncomfortable for most PFAs. *“A CEO? Why am I sitting here with a CEO? And a chairman of the board; vice‐president of this, vice‐president of that; supervisor of this – and I think, ‘What am I doing here?’”*

In spite of initial uncertainties, PFAs uniformly agreed that they were strongly encouraged to contribute and their perspectives were highly valued by the team. *“My opinion was just as important as the doctor's, and just as important as the CEO's.” “I was their equal, but I was also a patient…what I had to say mattered.”* PFAs consistently reported that genuine efforts were made to welcome and include them as part of the RPIW team, which quickly built trust and comfort. *“By giving you air time, by asking you questions, getting you engaged, using your direction.”* Expressions of “power with” were clearly fostered and led to new insights about roles and power of the PFAs. “*We have more latitude [than staff] because we're not employed [by the health care system]. We don't have a consequence or any intimidation. We can speak and share the things we'd like to see things as a patient.”*

PFAs enthusiastically supported fostering the power of front‐line providers, who were broadly seen by PFAs as having had relatively little voice in health‐care decision making in the past. RPIW teams involved front‐line providers. “*They're coming to the people who are actually doing the job…instead of coming up from some guy who has no idea what to do here, but he makes the decisions.”* Despite the recognition that changes resulting from RPIWs might prove uncomfortable for the health‐care providers, PFAs’ sense of duty to keep patients’ concerns foremost overrode concerns about the impact of changes to providers’ routines. *“We've got to remember it's not about [the health care professional], their little egos, who's doing more work, less work. It's all about the patient, the client.”* Not only were PFAs encouraged to voice their opinions, but the tangible evidence that their ideas were acted upon to make improvements within the scope of the RPIW's mandate supported “power within.” *“I was giving them some ideas about what would work out. **And** they were taking it and using it.”*

The key criteria for selecting effective PFAs for future initiatives were identified by participants as the capacity to act on the power available to the PFAs within the invited space. Having lived experience as a patient or family member within the health‐care system was seen as requisite to be able to contribute effectively to the discussion. “*If you've never experienced something that's gone terribly wrong with yourself, or a loved one you would have a very hard time saying this is what you need. You need the knowledge.”* PFAs had generally self‐selected involvement in particular RPIWs where they felt they had sufficient experience or knowledge to make a meaningful contribution.

For the very few participants who did report situations within an RPIW in which they felt disempowered by the attitudes of professionals (physicians, in particular), self‐confidence and trust (“power within”) in the process were critical. When “*people are not quite as welcoming, then it's my role to show them through my thoughtful contribution that I am a valuable player.”*

In terms of desired forms of future public engagement in quality improvement, most advised to continue with involvement in RPIW events, in spite of the recognized costs. *“I know it's expensive. But change is expensive. When it comes to people's lives, you can't put a price on that.”* Increasing public knowledge about the health‐care system was identified as fundamental for meaningful future engagement. *“It's great that all the healthcare providers know about [the health care system], but general public doesn't understand any of it.”* One PFA also recognized the reactive nature of the current RPIW process and suggested *“We could also be proactive and involve patient advisors or patients themselves in designing their own service… even before there's a problem go to them.”*

### 5.4 Outcomes of participation as a PFA

Facilitation of “power within” was a key outcome of participation for PFAs. Feelings of accomplishment dominated the discourse related to the outcomes of participation. *“For me, it's gratifying. It really is good to feel like I've helped make some of those changes.”* All PFAs clearly became more aware of the power they possessed within the health‐care system and further developed their skills at advocacy as a result of participation. *“Down the road, as we get more organized, we'll be interacting with more patients telling them what their rights are as a patient.” “[Before the RPIW], I did not speak up about certain things that you bet your life I speak up about now.”* Several PFAs identified a new and nuanced appreciation for the work of health‐care providers and the health‐care system as a whole. “*It changes your perspective…you get to appreciate things are sometimes not the way they seem.”*

Participation as a PFA also had personal, therapeutic benefits for some PFAs with long‐standing health conditions. *“This gave me something to focus on. Take me away from my problems.” “Having these different illnesses isn't a disability or an albatross around my neck. I can use it for good. I can sit at home and be depressed, or I can accept what I have and use it to help make things better.”* For others, participation as a PFA built social capital in the form of new personal connections, within both the health care and broader communities. Increased comfort with speaking in public instilled a new sense of confidence, allowing some PFAs opportunities to take part in media interviews and to take on efforts to mobilize more members of the public to participate as a PFA.

The time commitment meant the process was energizing for some PFAs, but very tiring for others. “*That week was exhausting. The end of the last day, I probably shouldn't have been driving home.”* The intensity of the process could be particularly difficult for PFAs who were living with health conditions, although the RPIW leaders did encourage PFAs to report when the process became overwhelming.

The only dissatisfaction voiced by PFAs related to the lack of follow‐up following the RPIW process. The sense of ownership and pride in their contribution was seriously eroded when there was little or no information provided about the progress of the changes they had helped to shape in the months following the event. “*If you going to tell me you're going to do follow‐ups, then do the follow‐ups. If you're not going to do them, don't say anything, because to me, you're letting us down.”*

## 6 DISCUSSION

**Figure 1:**
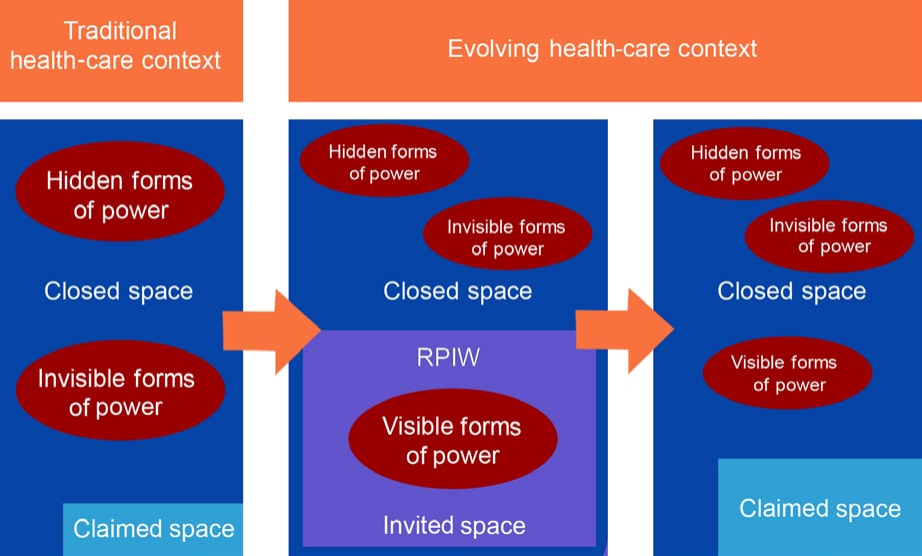
Patient Power in Evolution

The novel role of advisement on targeted health‐care redesign initiatives disrupted the conventional forms and spaces of power accorded to patients and families within this particular health‐care system in a time‐limited manner (Figure 1). Decision makers with formal power intentionally altered the form of power held by patients and families through the creation of an invited space within the RPIW. Given the deeply entrenched exclusion of patients and families from health‐care decision‐making spaces, this opportunity came to be viewed by PFAs as a genuine effort by those who governed the system to include the voices of consumers.

The goal of patient engagement within these activities was at the level of “collaboration”,^32^ but without final decision‐making power that characterizes the level of empowerment or shared execution. Gaventa's model^24^ suggests those who create the space retain power within it. Decision makers in this case retained complete control over the topics covered in the RPIWs, the format and organization of the events and the outcomes that were implemented. The opportunity for the public to contribute to setting future quality improvement agendas, rather than merely participating in the agendas of decision makers, would signal a welcome shift in forms and spaces of power.

According to Gaventa's model,^24^ however, potential for sustaining and creating new spaces depends on both strong mobilization from outside the space (ie, patients, families and the public) and on strong political will from decision makers to hold these spaces open. If engagement of the public in redesigning health care is to become normalized and embedded in the future, all stakeholders have critical roles to play. Increasing focus on the importance of patient and provider collaboration to codesign products and services is advocated as a primary mechanism to make patient‐centred care the new reality.^32^ Decision makers must create on‐going opportunities to welcome the input of and feedback from patients and families, while the public must assert its desire and willingness to contribute and engage with the health‐care system.

Efforts to support sharing “power with” PFAs were highly successful during the event itself, although this “power with” was time‐limited. For PFAs who had little or no on‐going contact with the team following the RPIW, the lack of opportunity to discover the outcomes of their efforts to improve quality underscored that the “power with” held by the PFAs had only been temporary. While the intent to continue to engage with PFAs on these initiatives may have been genuine, sufficient resources must be in place to allow communication to continue.

Participation as a PFA served as a potent demonstration to patients and families that sharing “power with” the health‐care system was both possible and desirable. While the PFAs in this study were activated and confident individuals, the traditionally closed spaces in which providers and bureaucrats made key health‐care decisions had previously excluded meaningful participation. Participation increased PFAs’ awareness and knowledge regarding operational and decision‐making aspects of the health‐care system, resulting in both new‐found confidence and skills for them to act as advocates. Some PFAs recognized the potential to independently create claimed spaces in the future to share their common issues and concerns with other patients and families and assert their power as consumers.

Achieving adequate representation of the population in coproduction efforts is limited by diverse interests and capacities on the part of patients.^7^ Participation in these RPIWs was limited to those who could afford the time and had sufficient resources to participate in this event. While recruitment materials stated that a key goal of this initiative was to obtain input from a diverse group of patients, forms and spaces of power served to restrict engagement. Inclusion of traditionally ‘hard to reach’ groups will require the creation of forms and spaces of power acceptable to these populations. The need for alternate forms of engagement for those with health, material or social disadvantages is predicated on that value that “the healthcare system cannot abandon patients who do not have the resources or expertise to partner effectively in coproducing good outcomes for themselves.”^7^ Health systems have a moral imperative to identify strategies for engagement and to support opportunities for continued debate about the focus and methods of involvement.^10^

Large‐scale transformation of health‐care systems with multiple missions and a broad array of stakeholders must, begin with incremental changes to group norms^33^ such as those that took place within the invited space of the RPIW. Provider perspectives on patient engagement make a significant contribution to the normalization of patient‐centred culture. Future research examining if, how and why providers value patient engagement will be an important contribution to the literature in this area. The evolution of patient engagement initiatives will increasingly be founded on the premise that user‐negotiated structures and processes must be on‐going and dynamic.^34^

### 6.1 Strengths and limitations

Use of Gaventa's theory of power^24^ provided conceptual underpinnings to our study of the experience of PFAs in quality improvement initiatives that can be built upon in future research. While all PFAs had participated in an RPIW within the previous year, variability in time elapsed since participation may have influenced recall to some extent. Studying the PFA experience immediately following the engagement experience may have promoted consistency of recall. Although our findings cannot be generalized to other settings as this was an investigation in a single Canadian province using a particular form of patient engagement, we believe that the study provided valuable insights into the role of power and into the perspectives of patients and families who participated in these workshops.

## 7 CONCLUSIONS

Acknowledging the role of power within health care, and the ways in which power differentials affect the relationships between patients, families, providers and decision makers, is pivotal to advancing patient‐centred care. Gaventa's^24^ theory served as a helpful framework with which to examine the forms and spaces of power for PFAs participating in extended quality improvement events and can be used to develop new strategies to incorporate patient and family voices into the design and evaluation of health initiatives. While the success of strategies to promote patient engagement are contingent on a host of factors within a given health‐care system (eg, public and funder health priorities, organizational cultures),^33^ patients and the public appear eager to embrace the opportunities and the changes in power that accompany the transformation to a more patient‐centred system.
